# Six new species of *Philiris* Röber, 1891 (Lepidoptera, Lycaenidae) from Papua New Guinea

**DOI:** 10.3897/zookeys.395.7110

**Published:** 2014-04-01

**Authors:** Chris J. Müller

**Affiliations:** 1 Papua Mining PLC, 5th Floor, 17 Hanover Square, London W1S 1HU, England. Address for correspondence: PO Box 3228, Dural, NSW 2158, Australia

**Keywords:** Taxonomy, Lepidoptera, Lycaenidae, Luciini, new species, Papua New Guinea

## Abstract

Six new species of the large lycaenid genus *Philiris* Röber, 1891 (*Philiris petriei* **sp. n.**, *Philiris bubalisatina* **sp. n.**, *Philiris baiteta* **sp. n.**, *Philiris radicala* **sp. n.**, *Philiris hindenburgensis* **sp. n.** and *Philiris parsonsi* **sp. n.**), from Papua New Guinea, are described and illustrated, as are the early stages of the former taxon, with *Litsea* sp. near *callophyllantha* K. Schum (Lauraceae) recorded as the larval food plant. The holotypes of all but the latter are deposited in the ANIC, with that of *P. parsonsi* located in the BMNH. The external facies and male genitalia of all new species are compared in detail to putative known related species, and the types of these, in nearly all cases, are also illustrated.

## Introduction

*Philiris* Röber, 1891 is one of the most speciose genera in the Australian region, where it is confined to Maluku, New Guinea, the Bismarcks and eastern Australia, with a concentration on the mainland of New Guinea. The genus was revised by Tite (1963), who recognised 56 species and later in an unpublished thesis by Sands (1981a) who added an additional 11 species in two separate publications (Sands 1979, 1981b) and placed the taxa into 21 species groups (validated by Parsons 1998), although he suggested that certain groups were ill-defined. Tite (1963) assumed a relationship of *Philiris* with *Candalides* Hübner, 1819 but Eliot (1973) showed that the former is positioned within the tribe Luciini Waterhouse & Lyell, 1914, together with several other genera, which are all essentially restricted to the Australian Region. *Philiris* was placed by Eliot (1973) in the *Hypochrysops* C. Felder section of the tribe. The two genera are closely related and their adult behaviour is generally similar. Sands (1981a) proposed that the genus *Parachrysops* Bethune-Baker, 1904 is a synonym of *Philiris* (validated by Parsons 1998). Parsons (1984, 1998) modified this nomenclatural arrangement of the genus based on a study of *Philiris* early stages and their food plants. The early stages of several *Philiris* species have been documented in the literature (e.g. Forbes 1977; Parsons 1984; Wood 1984; Müller 2000).

Unlike members of related genera, the undersides of many *Philiris* are relatively unmarked and generally similar, which was discussed by Tite (1963). However, most species have a characteristic pink, yellow, silver, grey or golden hue which is useful in the identification of species, as is the presence, including intensity, or absence of a black spot on the inner margin of the hindwing underside (Sands 1981a).

Morphology of the male genitalia, in particular the aedeagi, sociunci and valvae provides important characteristics for separating closely related species within Luciini (Tite 1963; 1969; Sands 1981a). These workers showed that vast differences are apparent in the shape of the valvae, including some species which exhibit asymmetry.

Members of *Philiris* inhabit altitudes between sea level and almost 2000 m, with many species in mainland New Guinea being restricted to the lower to mid-montane zones and being apparently absent from the lowlands (Parsons 1998). In this paper a number of new species from this altitudinal zone are introduced, largely as a result of systematic surveying in remote areas of Papua New Guinea in recent years.

Descriptions follow that of the numerical vein system.

## Materials and methods

Specimens were collected using long-handled nets and/or reared from early stages sleeved on their larval food plants. Adults and immature stages were photographed using a Nikon D300s Digital SLR Camera with a Nikon AF-S VR Micro-Nikkor 105 mm f/2.8G IF-ED Macro lens and Nikon R1C1 Close-up Kit Flashes Speedlites and Speedlights. Genitalia were photographed using the same camera with a Meiji Techno EMZ-5TR-P-FOI Trinocular Stereozoom Microscope, with OPTEK FL95E Fibreoptic Illuminator and twin arm optical fibre. Landscape photographs were taken using the above camera with an AF-S DX Nikkor 18-105 mm f3.5-5.6G ED VR Lens. Individual sliced genitalia images were concatenated using the software Helicon Focus 6.0 and edited in Adobe Photoshop CS6. Genitalia slides were photographed using a Nikon CoolScan ED5000 with modified slide scanner. Plates were designed in Adobe InDesign CS6.

### Abbreviations

AM Australian Museum, Sydney, Australia.

ANIC Australian National Insect Collection, Canberra, ACT, Australia.

BMNH British Museum (Natural History), London, England.

CJMC Reference collection of Chris J. Müller, Sydney, Australia.

EAPC Reference collection of Edward A. Petrie, Sydney, Australia.

UFL McGuire Collection, University of Florida, United States of America.

NARI National Agricultural Research Institute, Boroko, Port Moresby, Papua New Guinea.

## Taxonomy

### Philiris petriei

Müller sp. n.

http://zoobank.org/76578F35-0BE9-4C66-BEAB-94A69C8E6B72

http://species-id.net/wiki/Philiris_petriei

Figs 1–4 , 61 , 88–92 , 97

#### Type material.

**Figures 1–15.**
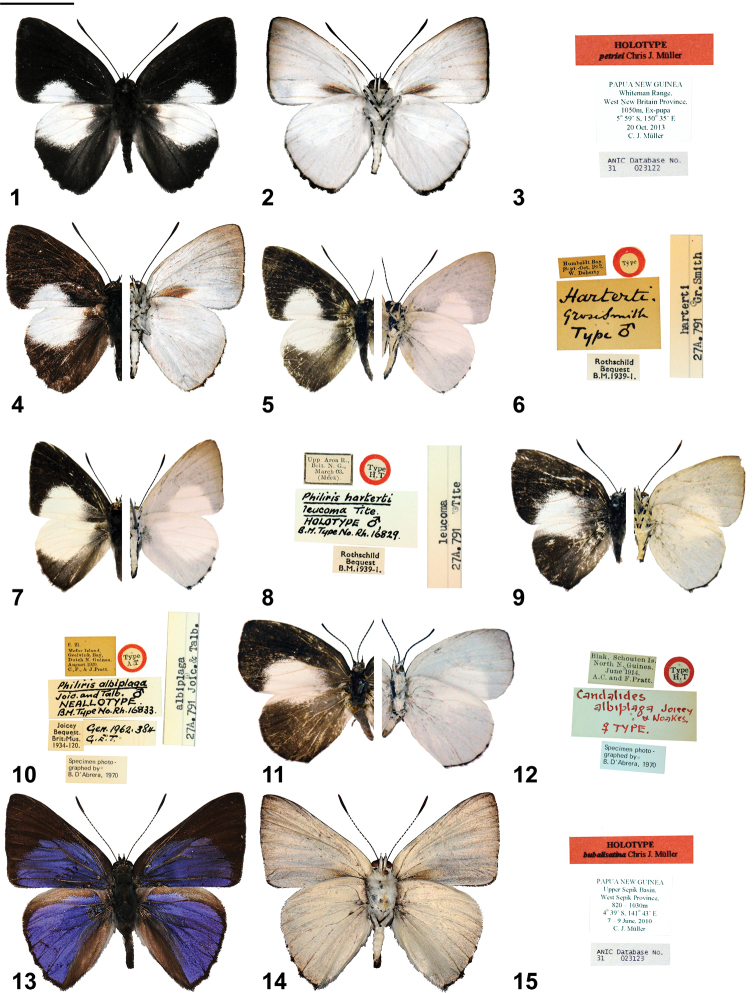
*Philiris* adults (left side upperside and right side underside, where halved) and label data. **1** *Philiris petriei* holotype ♂ upperside **2** *Philiris petriei* holotype ♂ male underside **3** *Philiris petriei* holotype ♂ label data **4** *Philiris petriei* paratype ♂ (halved) **5** *Philiris harterti harterti* holotype ♂ (halved) **6** *Philiris harterti harterti* holotype ♂ label data **7** *Philiris harterti leucoma* holotype ♂ (halved) **8** *Philiris harterti leucoma* holotype ♂ label data **9** *Philiris albiplaga* neoallotype ♂ (halved) **10** *Philiris albiplaga* neoallotype ♂ label data **11** *Philiris albiplaga* holotype ♀ (halved) **12** *Philiris albiplaga* holotype ♀ label data **13** *Philiris bubalisatina* holotype ♂ upperside **14** *Philiris bubalisatina* holotype ♂ underside **15** *Philiris bubalisatina* holotype ♂ label data. Scale bar = 10 mm.

Holotype ♂ (Figs 1–3): Papua New Guinea, Whiteman Range, West New Britain Province, 1050 m, Ex-pupa, 5°59'S, 150°35'E, 20 Oct, 2013, Chris J. Müller, genitalia dissected and held in vial pinned to specimen, pupal exuvia pinned to specimen (ANIC), Registration: ANIC Database No. 31-023122. Paratypes (8 ♂♂): 4 ♂♂ labelled Papua New Guinea, Whiteman Range, West New Britain Province, 950 m, 5°58'S, 150°29'E, 10–18 Dec, 2005, Chris J. Müller (2 ♂♂ BMNH; 1 ♂ NARI; 1 ♂ EAPC); 3 ♂♂ labelled Mt. Otto summit, West New Britain Province, 1320 m, 5°33'S, 150°24'E, 19–22 Dec, 2006, Chris J. Müller (2 ♂♂ CJMC; 1 ♂ AM); 1 ♂ labelled Bainings Mts., East New Britain Province, 1000 m, 4°38'S, 152°02'E, xii.2008, L. Wills leg. (UFL).

#### Diagnosis.

Males of *Philiris petriei* sp. n. are readily distinguished from other species in the genus. *Philiris petriei* sp. n. is a large species with a forewing length that surpasses that of its putative congeners, *Philiris harterti* (Grose-Smith, 1894) and *Philiris albiplaga* (Joicey & Talbot, 1916). *Philiris harterti* occurs widely in mainland New Guinea (nominate in northern and western NG mainland; subspecies *leucoma* Tite, 1963 in eastern NG mainland). The holotypes of both taxa are illustrated here-in (see Figs 5–8). *Philiris harterti* also occurs on Yapen Island, where it is known by ssp. *melanoma* Tite, 1963. *Philiris albiplaga* is restricted to the Schouten Islands (Biak and Mefor). Since the holotype is a female specimen (Figs 11, 12), Tite (1963) designated one of two known males of *Philiris albiplaga* as a neallotype (Figs 9, 10).

The shape of the fore wing in *Philiris petriei* is slightly rounded at the apex, whereas it is pointed in the other species, particularly so in *Philiris harterti*. The hind wing termen is weakly serrated near the tornus in *Philiris petriei*, unlike in related species and the cilia on the underside of the hindwing tornal area is continuously black, as in *Philiris albiplaga*, whereas the cilia are mostly white in *Philiris harterti* and black only at the vein terminals. The underside of the forewing in *Philiris petriei* bears a large dark basal patch, not present in either *Philiris harterti* or *Philiris albiplaga*. The frons in *Philiris petriei* are black (Fig. 97), while they are rusty red and brown in *Philiris harterti* (Fig. 98) and *Philiris albiplaga*, respectively. The white area on the forewing above is much more reduced in *Philiris petriei* than in *Philiris harterti* and *Philiris albiplaga*, bearing more resemblance to the pattern in *Philiris bicolor* (Bethune-Baker, 1904), a species with red frons similar to *Philiris harterti*. In *Philiris petriei*, the forewing white patch is restricted to the inner margin and does not extend beyond vein 2, only marginally extending beyond 1b, while in *Philiris harterti* and *Philiris albiplaga* this white area continues beyond vein 2 and reaches, or exceeds, vein 3 and the edge of the cell. No obvious variation has been noted in the type series of *Philiris petriei*, with all specimens similar in size and maculation.

The male genitalia of *Philiris petriei* (Fig. 61) also show a relationship to both *Philiris harterti* (Fig. 62) and *Philiris albiplaga* (Fig. 71). However, the genitalia of the former species are larger than both *Philiris harterti* and *Philiris albiplaga*, and the sociuncus is narrowly V-shaped dorsally, whereas it is rather rounded in *Philiris harterti* and broadly U-shaped in *Philiris albiplaga*. The valva is triangular-shaped at the base in *Philiris petriei*, whereas it is bulbous in *Philiris harterti* and with conspicuous median lobes in *Philiris albiplaga*. The phallus is long and slender in *Philiris petriei* (Fig. 61c) and the vesica is boat shaped at its apex, whereas the phallus of *Philiris harterti* (Fig. 62c) is comparatively short and squat and the apex of the vesica is bifurcated symmetrically. The phallus of *Philiris albiplaga* is not clearly defined in either slide mounts of the Mefor Island male specimens but appears to be heavily sclerotized and the vesica is tongue-shaped (Fig. 71c).

#### Description.

♂ (Figs 1–3, 97): Forewing length 17.5 mm, antenna 11.5 mm (holotype). Head, palpus, thorax and abdomen black dorsally, white ventrally, frons black with white eye ring; antenna shaft black, ringed weakly with white, apical half of club orange-brown ventrally; legs white with black areas on tibiae.

Fore wing termen slightly convex but straight between veins 2 and 4, inner margin slightly convex; upperside with ground colour black, a restricted triangular area of white in the median and postmedian area, extending from the inner margin to midway between veins 1b and 2 and from about one-third along the termen to approximately 3 mm from the termen at the tornus, white area suffused with light grey narrowly towards base, cilia black; underside uniformly white, with apical area and termen broadly suffused with brown, a large rhomboid-shaped patch of dark brown scales extending from base to median area, cilia black.

Hind wing slightly rounded, termen weakly serrated between veins 2 and 4; upperside with ground colour black, apical area broadly white and extending along costa to near base and to the middle of the hind wing, filling distal half of cell and discocellulars, cilia black except in apical area where they are white; underside uniformly white with weak brown suffusion broadly along termen, cilia black and longer in tornal area, white at apex.

♀: Unknown.

Male genitalia (Fig. 61): Vinculum and tegumen ring broadly oval, sociuncus broad, socii with lateral margin rounded, socii weakly separated by V-shaped sinus, saccus tapered posteriorly, brachium tapered dorsally and slightly hooked at apex; valva long, symmetrical, triangular-shaped at base and tapered apically; phallus with prezonal section approximately equal in length to postzonal section, slender, with vesica apically flanged.

#### Etymology.

This species is named in honour of Edward A. Petrie, Sydney, Australia, renowned for his expertise in Australian Lycaenidae, in particular their life histories.

#### Distribution.

New Britain Island, Papua New Guinea.

#### Ecology.

When compared with those of the related *Philiris harterti*, the flight behaviour of *Philiris petriei* males differs in that the latter species generally flies on mountain tops (e.g. Fig. 84) from 1100 hrs until just after midday, whereas *Philiris harterti* does not normally appear in its hill top territories until about 1330. Males of *Philiris petriei* fly high, usually above six metres above the ground, whereas those of *Philiris harterti* generally fly much lower, between 1–4 metres above the ground. There appear to be two broods annually for the life cycle of *Philiris petriei*, with adults flying in November and December and again in April and May. Conversely, adults of *Philiris harterti* fly throughout the year in mainland New Guinea.

Parsons (1998) recorded the life history of *Philiris harterti* on saplings of *Litsea callophyllantha* (Lauraceae). A pupal exuvia of a *Philiris*, presumed to be *Philiris petriei*, was located in the Whiteman Range, New Britain, by the author during January 2013 on a small plant of *Litsea* species. Some months later two eggs were located on the same plant and one was sleeved. Both eggs hatched after some days and developed very slowly through the winter months, reaching final instar during August. One of the larva pupated in late September (larval duration 121 days), emerging in early Oct (pupal duration 14 days). Owing to the lack of florescence (flowers and/or fruit), it has not been possible to identify the *Litsea* species on which the new species feeds, although similarly to *Litsea callophyllantha*, it appears to grow very slowly and remain as a sapling for years until an opening in the canopy creates an opportunity for the plant to flourish. The food plant of *Philiris petriei* is very different from the large leaved *Litsea guppyi* (F. Muell) F. Muell. Ex Forman., the food plant of *Philiris siassi* Sands, 1979 (Müller 2002), which flies in the same habitats as *Philiris petriei*.

The early stages of both *Philiris harterti* (Figs 93–96) and *Philiris petriei* (Figs 88–92) are exceptionally well camouflaged on their foodplant, with those of *Philiris petriei* even more so than the larvae and pupae of *Philiris harterti*. The early stages are quite distinct between the two taxa, with the pupa of the new species being much less speckled and with more irregular dark patches than in *Philiris harterti*. The anterior of the pupa of *Philiris petriei* lacks the very long setae present in *Philiris harterti*. The mature larvae of the two taxa differ in that that of *Philiris petriei* is brown while the larva of *Philiris harterti* is greenish and the setae are much coarser in the latter species. The early stages of *Philiris petriei* are described below.

Egg (Not illustrated): Approximately 0.5 mm diameter, white, domed, wider than high, intricately sculptured. Similar to those of other *Philiris* taxa examined.

First Instar (Not illustrated): c. 1.0 mm long, grass green, with long translucent setae.

Second Instar (Fig. 88): c. 4 mm long, 1.5 mm wide, deep green centrally with light green-brown margins, with long translucent setae.

Third Instar (Not illustrated): c. 7.0 mm in length, 1.5 mm wide, similar to second instar but with lateral margin brown rather than green.

Fourth Instar (Fig. 89): c. 10.0 mm in length, 2.5 mm wide, flattened, flanged laterally, centrally deep green with broad light brown margins, finely speckled with white, with long light brown setae on margins.

Final Instar (Fig. 90): c. 15 mm in length, 4.5 mm wide, flattened, flanged laterally, light red-brown, weakly speckled with white, with long light brown setae on margins.

Pupa (Figs 91, 92): 13 mm in length, 4.5 mm wide, light brown with dark brown irregular mottled patches, particularly on eyes and surrounding the wing cases, fine short setae (<1 mm long) anteriorly and on abdomen.

Similarly to the larva of *Philiris harterti*, the first two instars feed on the underside epidermis of the leaf of the food plant, sheltering concealed within these shallow depressions which become feeding scars on the foliage. The third instar larvae chews troughs from the stem of the food plant, within which it eventually rests, progressively increasing the size of the trough to accommodate the sheltering larva. At this stage the larva also chews right through the leaf, creating holes. Eventually, the larva pupates within the trough, where it is remarkably well concealed.

#### Remarks.

*Philiris petriei* may possibly be restricted to the island of New Britain within the Bismarck Archipelago. Substantial surveying of pristine habitats by the author in New Ireland from sea level to the highest elevations has not yielded any specimens of this taxon. The insect may also be confined to upland habitats, with all specimens taken above 950 m. It appears to be a rather rare species, possibly due to the scarcity of its larval food plant.

### Philiris bubalisatina

Müller sp. n.

http://zoobank.org/FAC04FF7-1FF1-4256-B386-53F2A17E3F58

http://species-id.net/wiki/Philiris_bubalisatina

Figs 13–15 , 63

#### Type material.

Holotype ♂ (Figs 13–15): “Papua New Guinea, Upper Sepik Basin, West Sepik Province, 4°39'S, 141°43'E, 820–1030 m, 7–9 June, 2010, Chris J. Müller, genitalia dissected and held in vial pinned to specimen, (ANIC), Registration: ANIC Database No. 31-023123. Paratypes (2 ♂♂): 1 ♂ labelled “Papua New Guinea, Upper Sepik Basin, West Sepik Province, 560 m, 4°40'S, 141°46'E, 8–12 Feb, 2010, C. J. Müller” (AM); 1 ♂ labelled “Papua New Guinea, Baiyer River, Western Highlands Province, 1190 m, 5°30'S, 144°10'E, 12–18 Nov, 2013, C. J. Müller” (CJMC).

#### Diagnosis.

*Philiris bubalisatina* is unlike any known species, its external facies showing affinities with the *Philiris marginata* (Grose-Smith, 1894), *Philiris fulgens* (Grose-Smith & Kirby, 1897) and *Philiris helena* (Snellen, 1887) groups. The wing shape of *Philiris bubalisatina* is highly acute, with a long pointed forewing and a remarkably elongate hind wing. The forewings are more exaggeratedly pointed than in *Philiris vicina* (Grose-Smith, 1898), *Philiris marginata* (holotypes; Figs 18, 19, 77 and 20, 21, 76, respectively) (both *Philiris marginata* group) and *Philiris fulgens* (holotype; Figs 22, 23, 78), even ‘subspecies’ *septentrionalis* Joicey & Talbot, 1916 (holotype; Figs 24, 25, 79). The fore wing upperside is a bright shining lilac-blue, which is more bluish on the hind wing and the dark margins are very broad, while that on the hind wing is of hairline thickness. The underside of *Philiris bubalisatina* is unique in *Philiris*, being a pale buff-cream, with a yellowish hue. Unlike other species with non-white undersides (essentially those in the *helena* group), e.g. *Philiris apicalis* Tite, 1963 (holotype; Figs 26, 27, 80 and subspecies *ginni* Müller; Fig. 28), that of *Philiris bubalisatina* is semi-glossy rather than matt. There is no black spot at the inner margin of the hind wing underside. There appears to be little variation in the type series of *Philiris bubalisatina*, although one paratype from the Upper Sepik has a slightly shorter fore wing length and the shape of the fore wing is slightly more convex than in the other specimens.

**Figures 16–30.**
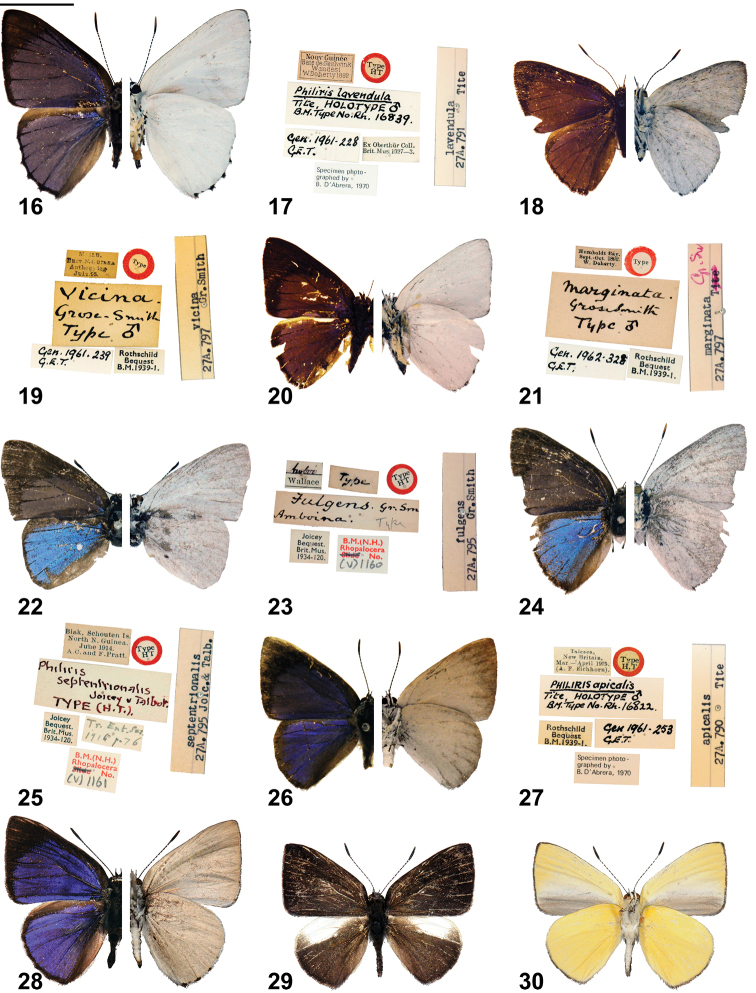
*Philiris* adults (left side upperside and right side underside, where halved) and label data. **16** *Philiris lavendula* holotype ♂ (halved, flipped horizontally) **17** *Philiris lavendula* holotype ♂ label data **18** *Philiris vicina* holotype ♂ (halved) **19** *Philiris vicina* holotype ♂ label data **20** *Philiris marginata* holotype ♂ (halved, flipped horizontally) **21** *Philiris marginata* holotype ♂ label data **22** *Philiris fulgens* holotype ♂ (halved) **23** *Philiris fulgens* holotype ♂ label data **24** *Philiris fulgens septentrionalis* holotype ♂ (halved) **25** *Philiris fulgens septentrionalis* holotype ♂ label data **26** *Philiris apicalis* holotype ♂ (halved) **27** *Philiris apicalis* holotype ♂ label data **28** *Philiris apicalis ginni* ♂ (halved) **29** *Philiris baiteta* holotype ♂ upperside **30** *Philiris baiteta* holotype ♂ underside. Scale bar = 10 mm.

The male genitalia of *Philiris bubalisatina* is also highly distinctive and does not closely resemble those of any known *Philiris*. The bulbous valvae with lateral appendages are unusual and are only otherwise shared with *Philiris lavendula* Tite, 1963 (holotype; Figs 16, 17, 75). However the sociuncus in the male genitalia of the latter species is deeply incised and the valvae are much more acute than in *Philiris bubalisatina*. *Philiris bubalisatina* and *Philiris lavendula* have dissimilar external facies, with the former species having a broad forewing upperside border with a shining lilac-purple ground colour and a buff-coloured underside, whereas *Philiris lavendula* has a much narrower upperside border, a matt purple ground colour and a white underside. The wing shape of the two taxa also differ markedly.

#### Description.

♂ (Figs 13, 14): Forewing length 20 mm, antenna 12 mm (holotype). Head, palpus and thorax dark grey dorsally, cream ventrally, abdomen dark grey dorsally, cream-buff ventrally, frons dark grey with white eye ring; antenna shaft black, ringed conspicuously with white between segments, apical half of club brown ventrally; legs cream with black areas on tibiae.

Forewing termen nearly straight, inner margin straight, apex pointed; upperside with ground colour black, a large area of shining purple-lilac extending from base to near end of cell and postmedian area to approximately 2 mm from termen at tornus, cilia black; underside uniformly glossy pale buff-cream with apical area suffused with dark scales, darker basal patch at inner margin, cilia narrowly black.

Hindwing elongated towards tornus, slightly produced near tornus at veins 2, 3 and 4; upperside shining purple-blue (more bluish than fore wing shining area) and narrowly bluish-white nearest to costa, termen narrowly (<1 mm) black, costa light brown above vein 7 and midway between cell and vein 8, merging with dark termen between veins 7 and 6, inner margin broadly brown to vein 1b, cilia black; underside uniformly glossy pale buff-cream with dark brown scaling near termen, cilia narrowly black, broader at tornus and at ends of veins 2, 3, and 4.

Male genitalia (Fig. 63): Vinculum and tegumen ring oval, tapered towards sociuncus, sociuncus rather broad, socii with lateral margin square-shaped, dorsally rounded, socii weakly separated by V-shaped sinus, saccus tapered posteriorly, brachium tapered dorsally and slightly hooked at apex; valva symmetrical, bulbous at base with long appendage stemming from lateral margin; phallus large, with zone of intricate cornuti in post-zonal section, vesica apically flanged.

♀: Unknown.

#### Etymology.

The name is a combination of the Latin word ‘bubalinus’, for the colour buff, reflecting the unusual pale yellowish-brown hue to the underside, and ‘satina’, which refers to the satin lustre to the underside.

#### Distribution.

West Sepik and Western Highlands Provinces, Papua New Guinea.

#### Ecology.

All specimens of *Philiris bubalisatina* were taken at, or just before, midday as they perched momentarily on foliage overhanging rapid torrents, several metres above the ground. The taxon has a remarkably rapid and robust, wide-ranging flight, which is rather Hesperiid-like.

#### Remarks.

Since it is difficult to accurately determine, the correct nomenclatural positioning of *Philiris bubalisatina* would be assisted by information about its life history and larval food plants. Several *Philiris* larvae were found in the general type locality area and some were reared to adult (e.g. *Philiris violetta* (Röber, 1926), *Philiris praeclara* Tite, 1963 and *Philiris harterti*). It is not known if larvae of the new species were present among individuals that were not able to be reared to adult due to time constraints. A molecular phylogeny of the genus *Philiris* will undoubtedly better resolve its taxonomic position.

### Philiris baiteta

Müller sp. n.

http://zoobank.org/E2CE34E9-1520-42A5-9E95-DAA8C6D2CBF3

http://species-id.net/wiki/Philiris_baiteta

Figs 29 –33 , 64

#### Type material.

**Figures 31–45.**
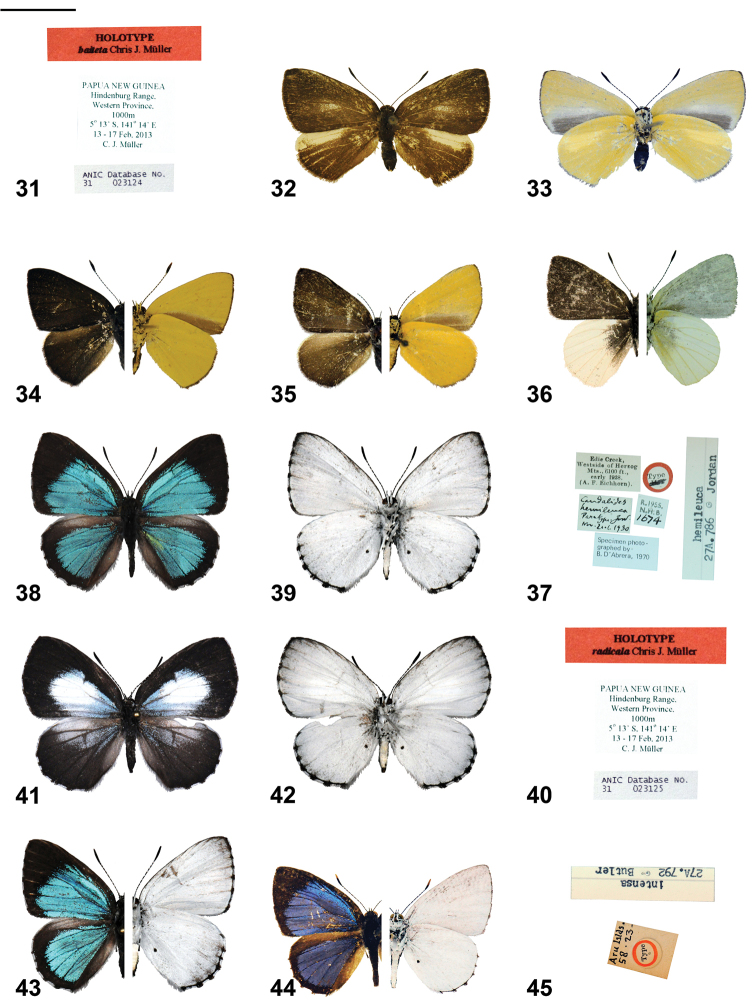
*Philiris* adults (left side upperside and right side underside, where halved) and label data. **31** *Philiris baiteta* holotype ♂ label data **32** *Philiris baiteta* paratype ♀ upperside **33** *Philiris baiteta* paratype ♀ underside **34** *Philiris hypoxantha* ♂ (halved) **35** *Philiris hypoxantha* ♀ (halved) **36** *Philiris hemileuca* holotype ♂ (halved, flipped horizontally) **37** *Philiris hemileuca* holotype ♂ label data **38** *Philiris radicala* holotype ♂ upperside **39** *Philiris radicala* holotype ♂ underside **40** *Philiris radicala* holotype ♂ label data **41** *Philiris radicala* paratype ♀ upperside **42** *Philiris radicala* paratype ♀ underside **43** *Philiris radicala* paratype ♂ (halved, flipped horizontally) **44** *Philiris intensa* holotype ♂ (halved) **45** *Philiris intensa* holotype ♂ label data. Scale bar = 10 mm.

Holotype ♂ (Figs 29–31): “Papua New Guinea, Hindenburg Range, Western Province, 1000 m, 5°13'S, 141°14’E, 13–17 Feb, 2013, Chris J. Müller, genitalia dissected and held in vial pinned to specimen, (ANIC), Registration: ANIC Database No. 31-023124. Paratypes (8 ♂♂, 1 ♀): 4 ♂♂ labelled the same as the holotype (1 ♂ AM, 1 ♂ BMNH, 2 ♂♂ CJMC); 3 ♂♂ labelled “Papua New Guinea, Baiteta, Madang Prov. 5°00'S, 145°44'E, 380 m, 6 August 1987, D.P.A. Sands (on loan to ANIC); 1 ♂ labelled the same as last but 31 July 1987 (on loan to ANIC); 1 ♀ labelled “nr. Oetakwa R., Snow Mts., Dutch N. G., up to 3500 ft., x. xii. 1910 (Meek)” (BMNH).

#### Diagnosis.

*Philiris baiteta* is a distinctive species that was previously confused with the related *Philiris hypoxantha* (Figs 34, 35). The type specimen of *Philiris hypoxantha* from south-western Papua Province could not be located but the description (in German) by Röber (1926) is detailed and he describes the wings above as ‘monotonously sepia-brown with low shine’ Röber (1926, p. 375, translated).

A single female of *Philiris baiteta* in the BMNH (Fig. 32, 32) was recognised tentatively as a ‘form’ of *Philiris hypoxantha* by Tite (1963) who also added comment that its significance could not be assessed until more material became available. This is obviously the true female belonging to *Philiris baiteta*.

*Philiris baiteta* is readily distinguished from *Philiris hypoxantha* by the large area of cream-white in the costal and subapical area of the hindwing upperside. Both wings above are uniformly brown in *Philiris hypoxantha*. The underside of *Philiris baiteta* is cream-yellow, with a broad cream border to the greyish area on the inner margin of the forewing. Conversely, in *Philiris hypoxantha*, the underside is bright yellow.

The unusual male genitalia of *Philiris baiteta* (Fig. 64), *Philiris hypoxantha* (Fig. 66) and *Philiris hemileuca* (Fig. 65) emphasise a close relationship of the three taxa, yet each show vast differences in their morphology, in particular the shape of the valva. In all three species the valva is sharply tapered to a spike anteriorly. In both *Philiris hemileuca* (Fig. 65b) and *Philiris baiteta* (Fig. 64b) an additional posterior dorso-lateral spike is present, which is much longer and more pronounced in the latter species than in *Philiris hemileuca*. The phallus of the three species also exhibit notable differences with *Philiris hypoxantha* bearing an apically enlarged vesica (Fig. 66c), which is bifurcated in *Philiris hemileuca* (Fig. 65c), yet rather abruptly terminated in *Philiris baiteta* (Fig. 64c). Parsons (1998) noted that the male genitalia of *Philiris hypoxantha* bear some resemblance to those of *Philiris vicina* (Fig. 77), especially in the shape of the valvae and aedeagus.

#### Description.

♂ (Figs 29–31): Forewing length 14 mm, antenna 8 mm (holotype). Head, palpus and thorax dark brown dorsally, white ventrally, abdomen dark brown dorsally, white ventrally, frons dark grey with white eye ring; legs white with black areas on tibiae; antenna shaft black, ringed conspicuously with white between segments, club wholly black.

Fore wing termen nearly straight, inner margin straight, apex pointed; upperside with ground colour dark uniform brown, cilia brown; underside pale yellow-cream, grading through white towards inner margin where the area between the inner margin and vein 2 and the cubitus are light grey-brown, cilia dark brown.

Hind wing slightly acute at tornus; upperside with ground colour dark uniform brown, a large cream-white apical area extending from base to beyond vein 6 into space 5 but not connected to termen; cilia dark brown except at apex where they are cream; underside uniformly pale yellow, cilia as in upperside.

Male genitalia (Fig. 64): Vinculum and tegumen ring rather rectangular, sociuncus rather broad, square-shaped, socii with lateral margin square-shaped, dorsally rounded, socii not obviously separated by sinus, saccus tapered posteriorly, brachium tapered dorsally; valvae symmetrical, bulbous dorsally at base but flat, boat-shaped laterally, with long toothed appendage ventro-posteriorly and a shorter appendage dorso-posteriorly; phallus with pre-zonal section approximately equal in length to post-zonal section; zone of compacted cornuti in post-zonal section, vesica apically flanged.

♀: (Figs 32, 33), Forewing length 14.5 mm, antenna 8 mm. Similar to male but larger, wings much more rounded. Forewing upperside with diffuse patch of cream white in median area between veins 2 and 4; forewing underside with brown area along inner margin not reaching termen. Hindwing upperside with cream-white patch slightly more extensive, reaching further into space 5.

#### Etymology.

This species is named after the locality in Madang Province where Dr Don Sands, Brisbane, Australia, collected part of the type series, also recognising its distinction from the related *Philiris hypoxantha*.

#### Distribution.

Western and Madang Provinces, Papua New Guinea; Snow Mountains (Papua), Indonesia.

#### Ecology.

Both *Philiris baiteta* (Fig. 87) and *Philiris hypoxantha* were collected around midday as they settled on foliage some metres above the ground over a rapid flowing stream in precipitous terrain (Fig. 86), together with a number of other *Philiris* species. Both species exhibited a rapid, fluttering flight.

#### Remarks.

*Philiris baiteta* appears to form a small group within *Philiris* also comprising *Philiris hemileuca* (holotype; Figs 36, 37) and *Philiris hypoxantha* (Figs 34, 35) and *Philiris baiteta* appears to fall midway between the two. All species have been taken in the Hindenburg Range area, Western Province, where *Philiris hemileuca* occurs above about 1500 m, while *Philiris hypoxantha* and *Philiris baiteta* have been taken together at around 1000 m. The latter two species have also been taken flying together elsewhere, in the Snow Mountains, where A. Meek even collected both species on the same day.

### Philiris radicala

Müller sp. n.

http://zoobank.org/EEF3D73A-0478-45A0-83B8-997BBECA6B9B

http://species-id.net/wiki/Philiris_radicala

Figs 38–43 , 67 , 68

#### Type material.

Holotype ♂ (Figs 38–40): “Papua New Guinea, Hindenburg Range, Western Province, 1000 m, 5°13'S, 141°14'E, 13–17 Feb, 2013, Chris J. Müller, genitalia dissected and held in vial pinned to specimen, (ANIC), Registration: ANIC Database No. 31-023125. Paratypes (7 ♂♂, 2 ♀♀): labelled the same as the holotype (1 ♂ AM, 1 ♂ BMNH, 1 ♂ NARI, 4 ♂♂ CJMC, 1 ♀ ANIC, 1 ♀ AM).

#### Diagnosis.

**Figures 46–60.**
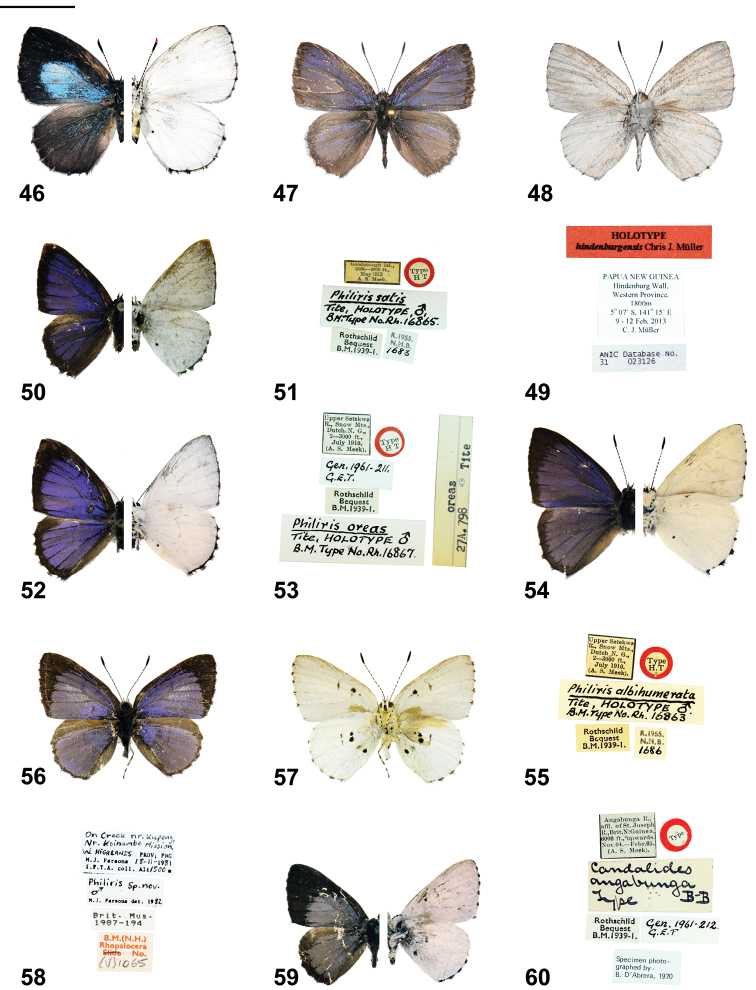
*Philiris* adults (left side upperside and right side underside, where halved) and label data. **46** *Philiris intensa* ♀ (halved) (Hindenburg Range, Western Province), **47** *Philiris hindenburgensis* holotype ♂ upperside **48** *Philiris hindenburgensis* holotype ♂ underside **49** *Philiris hindenburgensis* holotype ♂ label data **50** *Philiris satis* holotype ♂ (halved) **51** *Philiris satis* holotype ♂ label data **52** *Philiris oreas* holotype ♂ (halved) **53** *Philiris oreas* holotype ♂ label data **54** *Philiris albihumerata* holotype ♂ (halved) **55** *Philiris albihumerata* holotype ♂ label data **56** *Philiris parsonsi* holotype ♂ upperside **57** *Philiris parsonsi* holotype ♂ underside **58** *Philiris parsonsi* holotype ♂ label data **59** *Philiris angabunga* holotype ♂ (halved) **60** *Philiris angabunga* holotype ♂ label data. Scale bar = 10 mm.

*Philiris radicala* is unique within *Philiris*, with its very unusual colouration in the male upperside and matt white underside ground colour and dark border to the underside termen in both sexes. The taxon shows some relationship to *Philiris intensa* (Butler, 1876) (Holotype; Figs 44, 45 and female; Fig. 46), with which it flies in the Hindenburg Range, although the latter species is more commonly encountered below 500 m, while *Philiris radicala* flies between 700–1100 m. The male upperside of *Philiris intensa* is a deep, bright, shining sky blue, whereas that in *Philiris radicala* is an iridescent turquoise (green-blue). The dark border to both wings on the upperside of the male *Philiris radicala* is much broader than in *Philiris intensa* and the border of the forewing is straight, forming a line between the postmedian section of the costa and the tornus, while this border is curved in *Philiris intensa*. The female upperside of *Philiris radicala* bears very large white median patches on both wings, unlike *Philiris intensa* and the discocellulars of *Philiris radicala* are distinctly dark brown. On the underside of both sexes the ground colour is an unusual powdery matt white, the termen of both wings is heavily blackened, particularly at the vein ends, and dark scaling occurs in the subterminal area. These features are not present in *Philiris intensa*.

The male genitalia of *Philiris radicala* corroborates a relationship with *Philiris intensa*, with both possessing laterally pointed socii (more so in the latter species than in *Philiris radicala*, see Figs 69, 70) and similar aedeagi. Both taxa also bear triangular-shaped valvae, which are approximately equilateral in *Philiris radicala* but much longer and apically tapered in *Philiris intensa*. The valvae are slightly asymmetrical in *Philiris radicala* but symmetrical in the latter species.

**Figures 61–70.**
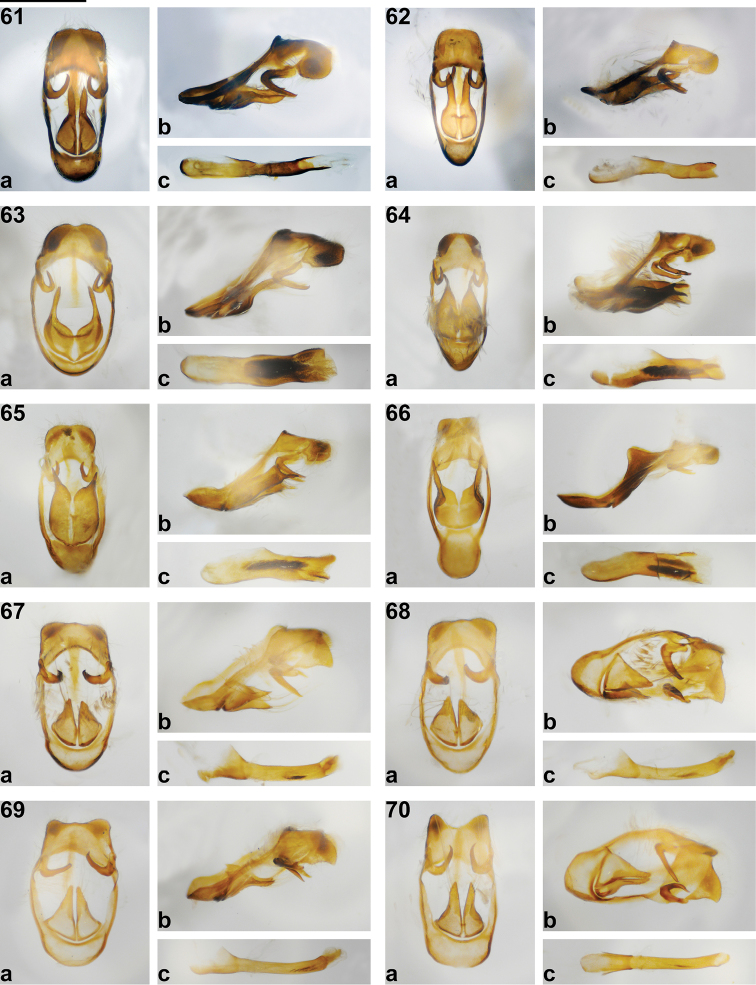
*Philiris* male genitalia (**a** genitalia in ventral view with aedeagus removed, **b** genitalia in lateral view, **c** aedeagus in lateral view). **61** *Philiris petriei* (Whiteman Range, West New Britain Province) **62** *Philiris harterti* (Nr. Wabo, Gulf Province) **63** *Philiris bubalisatina* holotype ♂ **64** *Philiris baiteta* (Hindenburg Range, Western Province) **65** *Philiris hemileuca* (Telefomin, West Sepik Province) **66** *Philiris hypoxantha* (Nr. Oetakwa River, Papua) **67** *Philiris radicala* holotype ♂ **68** *Philiris radicala* (Hindenburg Range, Western Province) **69** *Philiris intensa* (Bulolo, Morobe Province), **70** *Philiris intensa* (Nr. Wabo, Gulf Province). Scale bar = approx. 1 mm.

#### Description.

♂ (Figs 38–40): Forewing length 17 mm, antenna 9 mm (holotype). Head, palpus and thorax dark grey dorsally, white ventrally, abdomen dark grey dorsally, white ventrally, frons dark grey with white eye ring; legs white with black areas on tibiae; antenna shaft black, ringed conspicuously with white between segments, apex of club tipped with brown.

Fore wing termen slightly convex, inner margin very slightly bowed near base, apex slightly rounded; upperside bright shining turquoise, costa and termen broadly black, 2 mm wide at tornus but up to 5 mm wide at apex, the boundary between the dark border and shining turquoise area is straight between veins 2 and 8, cilia white but broadly black at vein ends, giving chequered appearance; underside matt white with apical area suffused with black scales, termen and vein ends distinctly black, cilia as in upperside.

Hind wing rounded; upperside bright shining turquoise, termen broadly black (2 mm wide), between inner margin and vein 1a dark brown-black, grading to light grey-brown basally, apex broadly black, costa broadly light grey, basally grey-brown, cilia white but broadly black at vein ends, giving chequered appearance; underside matt white, subterminal area narrowly suffused with black scales, termen and vein ends distinctly black, cilia as in upperside.

Male genitalia (Figs 67, 68): Vinculum and tegumen ring oval, enlarged towards sociuncus, sociuncus broad, socii with lateral margin pointed apically, dorsally sub-rounded, socii separated by slightly protruding sinus, saccus tapered posteriorly, brachium tapered dorsally and slightly hooked at apex; valva slightly asymmetrical, with left valva longer than left valva, valva equilateral triangle-shaped; phallus slender, with zone of intricate cornuti in post-zonal section, vesica with central ‘tongue’ apically.

♀ (Figs 41, 42): Forewing length (17 mm), antenna length (9 mm), antenna, head, palpus, thorax, legs and abdomen similar to male.

Fore wing termen slightly convex, inner margin very slightly bowed near base, apex slightly rounded; upperside dark brown-black, large area of white extending from base along inner margin to postmedian area and occupying about two-thirds of cell, margins of this pale patch suffused with powder blue, costal half of discocellulars heavily brown-black, intruding into pale area, cilia as in male; underside as in male.

Hind wing rounded; upperside dark brown-black, apical area broadly grey-white, cell and basal area along vein 1b grey-white with powder blue suffusion, inner margin and basal part of costa light grey-brown, discocellulars heavily brown-black, intruding into pale area, cilia as in male; underside as in male.

#### Etymology.

The name ‘radicala’ reflects the extraordinary colouration of the male upperside and overall divergent morphology of this species.

#### Distribution.

Western Province, Papua New Guinea.

#### Ecology.

Males of *Philiris radicala* were taken around midday and early afternoon as they settled at the tops of tall saplings, between 8–10 metres above the ground, in small clearings created by tree falls in very steep terrain. Females were recorded in similar areas, where they fed at the small white flowers of an unidentified tree. Conversely, males of *Philiris intensa*, flying at the same localities but generally at a lower altitude, were always seen to fly within 2–3 metres above the ground in areas of regrowth proximal to streams.

### Philiris hindenburgensis

Müller sp. n.

http://zoobank.org/5DE19383-BA6A-42EA-A2A2-FC8D199927C7

http://species-id.net/wiki/Philiris_hindenburgensis

Figs 47–49 , 72

#### Type material.

Holotype ♂ (Figs 47–49): Papua New Guinea, Hindenburg Wall, Western Province, 1800 m (5°07'S, 141°15'E), 9–12 Feb, 2013, Chris J. Müller (ANIC), Registration: ANIC Database No. 31-023126. No Paratypes.

#### Diagnosis.

*Philiris hindenburgensis* is a small species with rounded wings that is unique among those species in the genus with predominantly purple-blue uppersides to the males, in bearing a very broad dark border to the costa and inner margin of the hindwing upperside where the purple-blue is essentially restricted between veins 2 and 6. The broad forewing border that is parallel to the termen is also a feature of the males of *Philiris satis* Tite, 1963 (Holotype; Figs 50, 51, 81), *Philiris oreas* Tite, 1963 (Holotype; Figs 52, 53, 82) and *Philiris albihumerata* Tite, 1963 (Holotype; Figs 54, 55, 83). However, these taxa all have glossy white undersides with a large, prominent black spot on the inner margin of the hindwing underside. In *Philiris hindenburgensis*, the underside is a light grey-white and the spot on the inner margin is merely represented as a barely recognisable brown smear.

**Figures 71–83.**
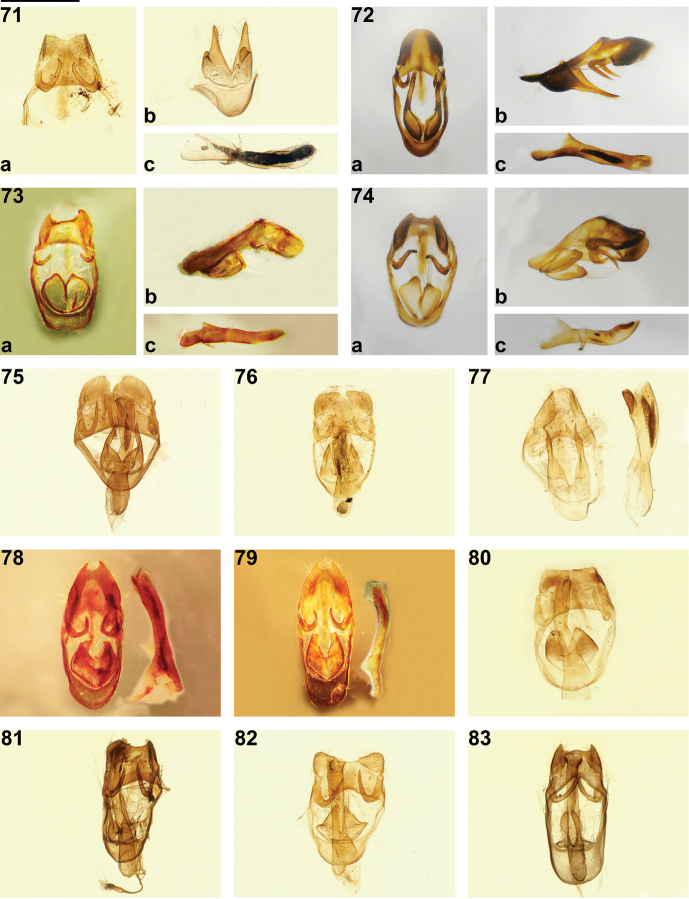
*Philiris* male genitalia. **71** *Philiris albiplaga* (Mefor Island, Papua) (**a** sociuncus in ventral view, **b** valvae in ventral view, **c** aedeagus in lateral view) **72** *Philiris hindenburgensis* holotype ♂, (**a** genitalia in ventral view with aedeagus removed, **b** genitalia in lateral view, **c** aedeagus in lateral view) **73** *Philiris parsonsi* holotype ♂, (**a** genitalia in ventral view with aedeagus removed, **b** genitalia in lateral view, **c** aedeagus in lateral view) **74** *Philiris angabunga* ♂ (Fane, Central Province) (**a** genitalia in ventral view with aedeagus removed, **b** genitalia in lateral view, **c** aedeagus in lateral view) **75** *Philiris lavendula* holotype ♂ genitalia in ventral view **76** *Philiris marginata* holotype ♂ genitalia in ventral view **77** *Philiris vicina* holotype ♂ genitalia in ventral view with aedeagus at right **78** *Philiris fulgens* holotype ♂ genitalia in ventral view with aedeagus at right **79** *Philiris fulgens septentrionalis* holotype ♂ genitalia in ventral view with aedeagus at right **80** *Philiris apicalis* holotype ♂ genitalia in ventral view **81** *Philiris satis* holotype ♂ genitalia in ventral view **82** *Philiris oreas* holotype ♂ genitalia in ventral view **83** *Philiris albihumerata* holotype ♂ genitalia in ventral view.

The male genitalia of *Philiris hindenburgensis* are highly distinctive and do not resemble those of any known *Philiris* species. The sociuncus is long and tapered such that the socii are not obviously separated and the lateral margin of the socii is concave. The valvae in *Philiris hindenburgensis* are most unusual, with long, slightly asymmetric appendages stemming from the lateral margin of the bulbous base.

#### Description.

♂ (Figs 47–48): Forewing length 15.5 mm, antenna 8.5 mm (holotype). Head, palpus and thorax dark grey dorsally, light grey ventrally, abdomen dark grey dorsally, light grey ventrally, frons dark grey with white eye ring; legs light grey with black areas on tibiae; antenna shaft black, ringed conspicuously with white between segments, apex of club brown.

Fore wing termen slightly convex, inner margin very slightly bowed in middle, apex slightly rounded; upperside dull frosty purple-blue, termen broadly dark brown-black and of even width (1.5 mm wide), cilia dark brown black; underside uniformly light grey-white, a small dark brown basal patch near inner margin, cilia light grey but dark brown-black at vein ends.

Hind wing rounded; upperside dull frosty purple-blue, costa and inner margin very broadly dark brown so that purple area is, with the exception of a few bordering purple scales, restricted between veins 2 and 6, termen broadly dark brown (approximately 1.5 mm wide), cilia light grey-white but dark brown at vein ends; underside uniformly light grey-white, a very obscure small brown spot between veins 1a and 1b approximately one third the distance from the base to the tornus, cilia light grey-white, dark brown-black at vein ends.

Male genitalia (Fig. 72): Vinculum and tegumen ring long, tapered posteriorly towards sociuncus, sociuncus narrow and rounded, socii with lateral margin pointed apically, concave in middle, dorsally socii unseparated by sinus, saccus tapered posteriorly, brachium long and tapered dorsally; valva slightly asymmetrical, with left valva longer than right valva, valva bulbous at base, with a long appendage stemming from lateral margin and tapering apically; phallus with large median zone of intricate cornuti, vesica with dorsal flange apically.

♀. Unknown.

#### Etymology.

This species is named after the type locality, the monumental Hindenburg Wall.

#### Distribution.

Western Province, Papua New Guinea.

#### Remarks.

Few species of *Philiris* occur at high altitude and in the Hindenburg Wall area at 1800 m (Fig. 85) and above, the only species recorded by the author, besides *Philiris hindenburgensis*, were *Philiris biplaga* Sands, 1981 and *Philiris montigena* Tite, 1963, all of which were recorded proximal to streams during rare periods of strong sunshine.

**Figures 84–98.**
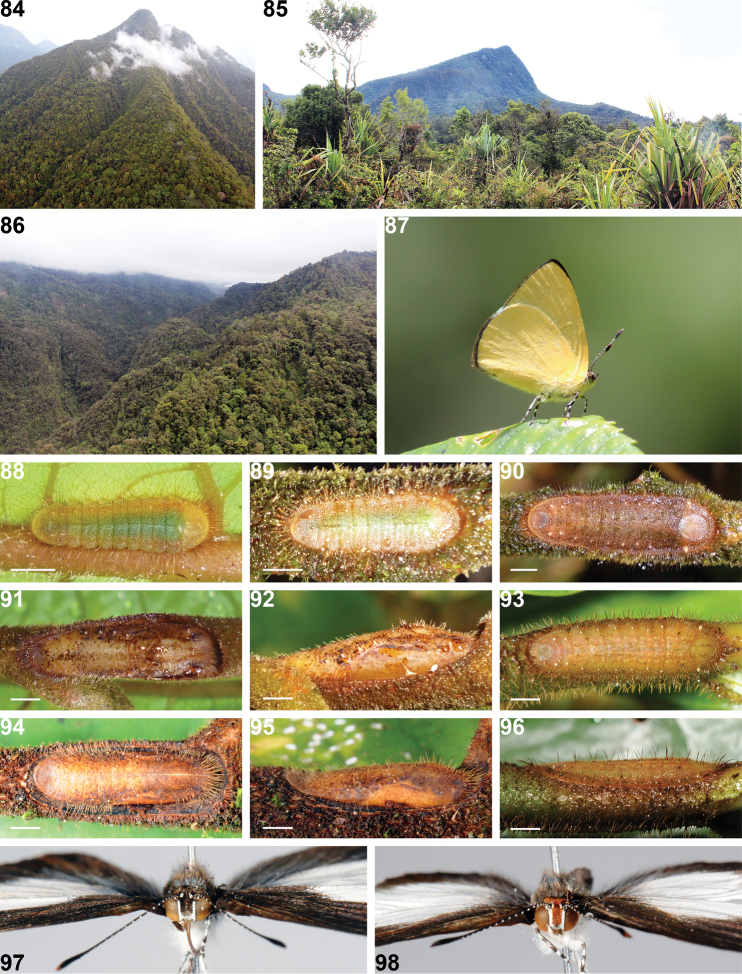
*Philiris* habitats, live adults and early stages. **84** Mt. Otto, West New Britain Province–typical habitat of *Philiris petriei* **85** Hindenburg Range 1800 m, Western Province–type locality of *Philiris hindenburgensis* **86** Hindenburg Range, approx. 1000 m, near type locality of *Philiris baiteta* and *Philiris radicala* **87** *Philiris baiteta* male perched in territory (Hindenburg Range) **88** *Philiris petriei* second instar larva in dorsal view **89** *Philiris petriei* fourth instar larva in dorsal view **90** *Philiris petriei* final instar larva in dorsal view **91** *Philiris petriei* pupa in dorsal view **92** *Philiris petriei* pupa in lateral view **93** *Philiris harterti* final instar larva in dorsal view **94** *Philiris harterti* pupa in dorsal view **95** *Philiris harterti* pupa in lateral view **96** *Philiris harterti* final instar larva in lateral view **97** *Philiris petriei* adult male frons **98** *Philiris harterti* adult male frons. Scale bar = 1 mm (Fig. 88), = 2 mm (Figs 89–96).

### Philiris parsonsi

Müller sp. n.

http://zoobank.org/92B7442D-AE55-430F-97EF-D716A2FC17D9

http://species-id.net/wiki/Philiris_parsonsi

Figs 56–58 , 73

Philiris sp. c (Parsons 1998, p. 378, Plates 53, XII, XXVI)

#### Type material.

Holotype ♂ (Figs 56–58, 73): “On Creek nr. Kupeng, Nr. Koinambe Mission, W. Highlands Prov; PNG, M. J. Parsons, 18-11-1981, I. F. T. A. coll. Alt 1500m”, “Philiris sp. nov. ♂, M. J. Parsons det. 1982”, “Brit. Mus. 1987-194”, B.M.(N.H.) Rhopalocera No. (V) 1065”. No Paratypes.

#### Diagnosis.

*Philiris parsonsi* is a distinctive species, presently known only from the unique male holotype, which Parsons (1998) illustrated as an undescribed species. He compared the external facies and male genitalia to *Philiris angabunga* Bethune-Baker, 1908 (Holotype; Figs 59, 60), within the *Philiris refusa* (Grose-Smith, 1894) group. However, the shape of the forewing and the colouration and maculation of both wing surfaces are unlike any of the described species within that species group. The forewing of *Philiris parsonsi* is more elongated than in other species and the dark border is much narrower, with the exception of *Philiris biplaga* and *Philiris pagwi* Sands, 1979, which have borders parallel to the termen and broader at the tornus, respectively, while that in *Philiris parsonsi* is widest at the apex. The upperside ground colour in *Philiris parsonsi* is an unusual pale lilac colour and there are white scales present in the median area of the forewing, not present in other species. The underside configuration of black spots is more complex than in other species and bears a total of ten individual markings, whereas a maximum of five spots are present in any other species (that for *Philiris maculata* Sands, 1981).

The male genitalia of *Philiris parsonsi* (Fig. 73) is also distinctive, the tegumen ring being very squat and squared dorsally when compared with that of *Philiris angabunga* (Fig. 74). The socii in *Philiris parsonsi* are widely spaced and the valvae are smaller than those of *Philiris angabunga* and rounded, whereas those of *Philiris angabunga* are triangular-shaped.

#### Description.

♂ (Figs 56–57): Forewing length 13.5 mm, antenna 7 mm (holotype). Head, palpus and thorax dark grey dorsally, white ventrally, abdomen dark grey dorsally, white ventrally, frons grey with white eye ring; legs white with black areas on tibiae; shaft black, ringed conspicuously with white between segments, apex of club brown.

Fore wing termen very slightly convex, inner margin very slightly bowed near base, apex slightly rounded; upperside pale lilac-blue, a scattering of white scales in median area, largely concentrated, but not restricted to, the area between veins 2 and 4, apical area broadly dark brown-black, border 5 mm wide at apex, narrowing evenly to meet tornus, cilia dark brown; underside dull white, cell with a large black spot (approximately 1.5 mm wide) about two thirds from base, two smaller obscure brown-black spots surrounding this spot, slightly closer to base and junction of cubitus with vein 3, discocellulars black, forming an elongated spot, cilia white, dark brown-black at vein ends.

Hind wing rounded; upperside pale lilac-blue, costa and inner margin dark, termen very narrowly dark brown (hairline thickness), cilia dark brown; underside dull white, two large (approximately 1.5 mm diameter) black spots in cell, about two thirds from base, a much smaller spot close to upper spot on basal side, a large black spot between veins 1a and 1b and another obscure dark brown spot of similar size between veins 1b and 2, both spots about one third from base, discocellulars black, forming an elongated spot, cilia as in forewing underside.

Male genitalia (Fig. 73): Vinculum and tegumen ring approximately rectangular-shaped, expanded at sociuncus, sociuncus broad, socii with lateral margin rounded, dorsally socii widely separated by U-shaped sinus, saccus short and squared dorsally, brachium short and abruptly terminated; valva slightly asymmetrical, with right valva larger than left valva, valva rounded with a small protrusion apically; phallus with pre-zonal section shorter than post-zonal section, tapered apically.

♀. Unknown.

#### Etymology.

Named after the collector of the holotype, Dr Michael Parsons, California.

#### Distribution.

Western Highlands Province, Papua New Guinea.

#### Remarks.

The unique holotype was taken in a creek by Parsons (pers. comm. 2013), flying together with *Philiris angabunga*. Although not listed on the specimen label, Parsons (1998) noted that the creek is known as Wara [Tok Pisin for River] Pimbi.

## Discussion

The distinctive black and white colour of both sexes of the species in the *Philiris harterti* species-group was noted by Sands (1981a). *Parachrysops* was originally erected for *Philiris bicolor* based on its red frons and wing venation but Sands (1981a) proposed that *Parachrysops* be subsumed within *Philiris* based on the two fore-mentioned characters being shared with *Philiris harterti*. *Philiris petriei* is very unusual within the group in that it possesses black frons, as opposed to the typical red frons.

Although Sands (1981a) proposed that *Philiris hypoxantha* and *Philiris hemileuca* be placed in their own species group, Parsons (1998) suggested, due to the colour above, in particular *Philiris hemileuca*, a relationship to the *harterti* species group.

There were two syntypes of *Philiris hemileuca* in the original description by Jordan (1930), described as *Candalides hemileuca*. The lectotype designated by Parsons (1998) is in poor condition when compared to the other syntype.

The genitalia slide preparation of the lectotype (then yet to be designated) by Bennett in 1955 for the work of Tite was significantly distorted (examined by the author) and was not illustrated by Tite (1963). Interestingly, Jordan (1930), in his description of *Philiris hemileuca*, well-illustrated the genitalia of this species, only then known by the two syntypes, of which only one (later designated the lectotype by Parsons (1998) was dissected. Therefore, Jordan obviously made drawings from the genitalia before it was mounted by Bennett. Jordan (1930) particularly noted the apically teethed claspers (valvae) of *Philiris hemileuca*.

This work has further high-lighted areas of butterfly endemism in Papua New Guinea. In particular, expeditions within the mountains comprising the central cordillera of New Britain Island have yielded other recent noteworthy discoveries (Müller 2013; Müller and Wills 2013 and references there-in). The Hindenburg Range and Star Mountains in Western Province also host a number of recently described taxa (Lachlan 1999, 2000) and the Upper Sepik Basin supports a number of distinctive butterfly taxa, yet to be described (C. Müller, unpublished).

## Supplementary Material

XML Treatment for Philiris petriei

XML Treatment for Philiris bubalisatina

XML Treatment for Philiris baiteta

XML Treatment for Philiris radicala

XML Treatment for Philiris hindenburgensis

XML Treatment for Philiris parsonsi
